# Exploring the ALS Multistep Model

**DOI:** 10.3390/brainsci16020236

**Published:** 2026-02-18

**Authors:** Andrew Eisen

**Affiliations:** Division of Neurology, University of British Columbia, Vancouver, BC V6T 1Z4, Canada; eisen@mail.ubc.ca

**Keywords:** amyotrophic lateral sclerosis, multistep model, environment, neurodevelopment, aging–senescence

## Abstract

**Highlights:**

**What are the main findings?**

- Combined environmental exposures act as modifiers to induce new steps in ALS.
- The multistep model encourages rethinking of therapeutic strategies in ALST.

**What are the implications of the main findings?**

- ALS may be preventable by protecting neurodevelopment and using anti-aging agents.
- Neurons and glia ecosystem are rescuable.

**Abstract:**

ALS is a multistep disease, in which (epi)genetic, environmental, and age-related processes, including senescence, converge over decades to reduce resilience resulting in self-sustaining symptomatic disease. The multistep model visualizes five to six impactful events in sporadic ALS, but fewer in those carrying high-penetrance mutations, such as SOD1, FUS, or C9orf72 expansions. The timing, duration, and cumulative effects of specific steps are presumed to have individual variability but, the steps themselves are inferred since they have not been observed and remain agnostic as to biological identity. Nevertheless, the model gives an opportunity to integrate genetics, aging, environmental exposures, and systems-level vulnerability into a single framework. Acting as step modifiers, environmental exposures including trauma lower the threshold for step acquisition, accelerate the accumulation of steps, influence the anatomical site of disease onset, and unmask preclinical disease. Because ALS emerges from the gradual collapse of multiple layers of biological robustness, tackling a single pathway will be insufficient and the multistep model forces a reconsideration of therapeutic timing and strategies. Protection against early-life insults, anti-aging, and anti-senescent therapies may curtail step accumulation preventing ALS from exceeding threshold and disease manifestation.

## 1. Introduction

ALS is a multifactorial, multistep disease, in which (epi)genetic, environmental (the exposome), and age-related processes, including senescence, converge over decades to induce clinically overt disease [1,2,3,4]. The multistep model, based on a linear relationship between the log incidence of ALS and the log age of onset, proposes that ALS requires the sequential accumulation of approximately five to six impactful “hits”. It provides an elegant explanation for initiation of ALS during the neonatal period [2,5]. Fewer steps are required in individuals carrying high-penetrance mutations such as SOD1, FUS, or C9orf72 expansions, and more steps in sporadic disease, possibly related to ALS phenotype [6,7,8,9,10,11,12]. The multistep process is probably applicable to all neurodegenerative disorders [13]. It is stochastic in nature, and the duration or repetition (cumulation) of specific steps in any given individual are unknown. For sporadic ALS there is an interplay of numerous small effect genes, some of which have been identified [14], and a broad array of possible environmental events occurring within the total exposome over a human lifetime [15]. The multistep hypothesis has been accepted for over a decade, but a recent study questions its validity [16], contending that age dependency of ALS fits better with the classic gene–time–environment hypothesis that posits that ALS onset is driven by the interaction between genes and environmental exposures over the life course as an accumulation that is more or less continuous, which is non-stepwise [16]. A significant advantage to the multistep model is the potential to apply interventional strategies even without knowledge of specific triggers preventing a “tipping point” for ALS to become clinically overt.

**Figure 1:**
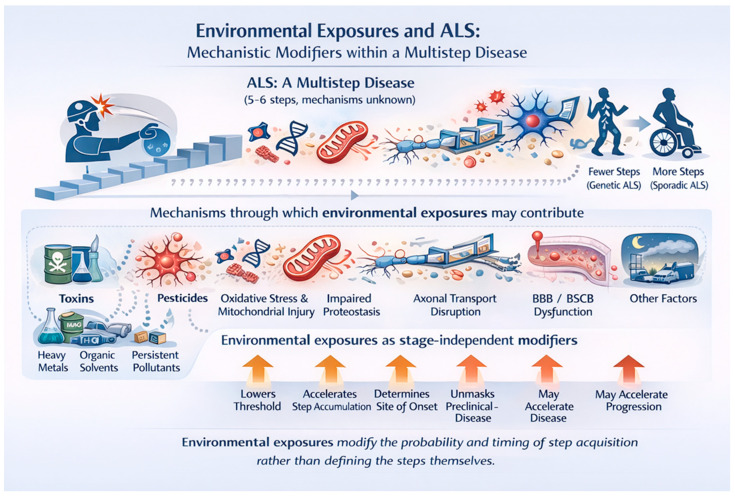
Amyotrophic lateral sclerosis (ALS) is conceptualized as a multistep disease requiring the accumulation of approximately five to six pathogenic events (“hits”), the precise biological nature and order of which remain unknown. Individuals with high-penetrance genetic variants require fewer steps to reach clinical disease, whereas sporadic ALS requires the accumulation of more steps over time. Environmental exposures—including trauma, pesticides, heavy metals, organic solvents, persistent organic pollutants, air pollution, micro- and nanoplastics and others (the ALS exposome), do not define these steps but influence disease risk through multiple convergent biological mechanisms. Shown are key mechanisms implicated in ALS pathogenesis that may be modified by environmental exposures, including neuroinflammatory priming, oxidative stress and mitochondrial injury, impaired proteostasis and protein handling, disruption of axonal transport, and dysfunction of the blood brain and blood spinal cord barriers. Acting as stage-independent modifiers, environmental exposures may lower the threshold for step acquisition, accelerate the accumulation of pathogenic steps, influence the anatomical site of disease onset, unmask preclinical disease, and potentially accelerate disease progression. This framework reconciles epidemiological and biological data by positioning environmental exposures as probabilistic modifiers of disease timing and expression rather than deterministic causes of ALS.

This opinion paper posits that the inconsistencies associated with many ALS environmental studies arise from a conceptual error. Their impacts have generally been evaluated as “causative” when variable unknown combinations of agents act as a mechanistic modifier within the ALS multistep process (Figure 1). Viewed this way, they lower the vulnerability of the neuronal and glial ecosystem increasing the probability of new steps and may also accelerate step accumulation. It has been calculated that for ALS there are between 6.7 and 13.7 years/step, underscoring the extended period before the disease becomes clinically overt [13]. Specifically considered are possible steps, how environmental exposures may act as mechanistic modifiers within the multistep model of ALS, and some strategies and potential therapies that prevent step accumulation.

### 1.1. Comparing the Multistep Versus Continuous Gene–Environment Models

For ALS two mathematical models have been proposed to describe the overall disease course. Bridging the gap between abstract mathematical theory and biological reality, approaches based on “best fit” is often limited and therefore controversial. The continuous gene–environment model predicts a “sliding scale” of a cumulative genetic–environmental risk for ALS, encompassing gradual fading of cellular resilience. The multistep model argues that ALS is the result of a discrete sequence of biological failures, only becoming clinically overt when the final step is complete. The multistep model was derived from population-level incidence curves and demonstrates that ALS arises from the probabilistic accumulation of several pathogenic events over time. It was adapted from cancer research (the Armitage–Doll model) and posits that motor neuronal degeneration requires a series of discrete molecular or cellular changes [6,8,17]. The steps are stochastic and inferred since they have not been observed and remain agnostic as to biological identity. Neither model needs be mutually exclusive; nevertheless, the multistep model gives an opportunity to integrate genetics, aging, environmental exposures, and systems-level vulnerability into a single framework that is potentially preventable or modifiable at given step intervals. This may prevent ALS from ever becoming clinically overt.

The multistep model likely explains why a disease with similar causative genetic mutations (like C9orf72) manifests at different ages and be phenotypically variable, exemplified by the presence or otherwise of frontotemporal dementia [18,19]. In a continuous (non-step) model, the onset would be more uniform. The multistep model indicates that even with a genetic defect at birth (Step 1), other independent events must occur for the disease to declare. Individual genetic background plays a significant role likely implicated in early steps. The presence of causative genes (SOD1, FUS, or C9orf72) requires fewer steps, whereas in sporadic ALS (>85% of cases), harboring numerous small-effect risk genes [20,21,22,23] requires up to 6 or 7 further steps for clinical manifestation. Many complex disorders, like ALS, are impacted by the interplay of genetic and environmental factors [24]. An individual’s genetic and epigenetic makeup influences the response to environmental exposures, which in turn impacts rate and number of step accumulations.

### 1.2. Biological Interpretation of Steps

It would be ideal to identify specific biological mechanisms that initiate individual steps allowing them to be blocked. This might eventually be plausible for those harboring “causative genes”. But other primarily non-genetic mechanisms are too variable and interdependent. For example, spontaneous mutations occurring throughout life may implicate a subset of neurons creating a vulnerable core within the brain or spinal cord that are more prone to a host of subsequent insults [25]. Exposure to toxins, heavy metals or physical trauma can impact epigenetic switches so DNA methylation may permanently silence genes responsible for a host of both positive and negative functions with downstream effects [26,27]. As a result, the neuron loses its ability to “reset” after stress, completing another step toward the threshold for disease. ALS is a “non-cell autonomous” disease, and a critical step may involve astrocytes and/or microglia within the neuronal ecosystem, which can initiate secretion of pro-inflammatory cytokines, setting the stage for chronic inflammation [28,29]. Axonal transport systems are critical to normal functioning and are particularly vulnerable in the motor system’s lengthy axons critical [30,31]. Microtubule dysfunction disrupts axonal integrity causing a breakdown in the transport of essential nutrients to the periphery and clearance of toxic waste, resulting in step progression [32].

### 1.3. Non-Genetic Early-Life Steps

Initial steps may occur in utero, the perinatal period and during early childhood development. During these vulnerable periods, the neuro-immune system is developing, driven largely by microglial maturation [33,34,35]. Abnormalities can induce a baseline shift in the intrinsic vulnerability of cortical and spinal motor neurons, and their supporting microglia, astrocytes, and endothelial cells, so they become less able to tolerate stress associated with neurodevelopment and its exposome [36,37,38]. Even if mild and asymptomatic, changes may persist and become largely irreversible, causing early-life inflammation, metabolic stress, developmental wiring anomalies, and energy demand mismatches, much of which is programmed through the developing microbiome and gut–brain axis [33,39]. Toxic environmental exposure during early life can induce persistent microglial priming, altering neuroimmune development [40,41], and epigenetic reprogramming [42,43,44], rendering the nervous system hyper-responsive lowering the threshold for subsequent injury and addition of new steps [45,46], and impact later life aging-related decline, senescent cell accumulation, and neurodegeneration [47,48,49].

### 1.4. Additional Steps

**Figure 2:**
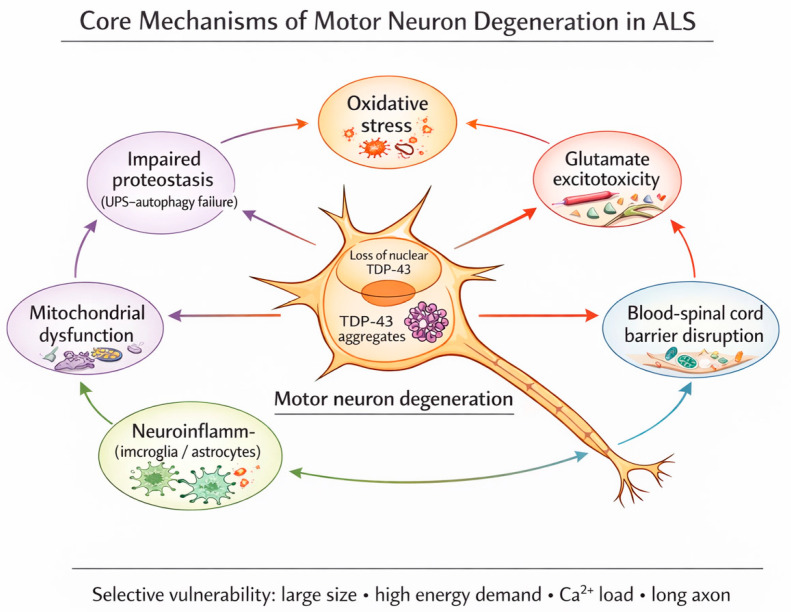
Interacting cascades in the pathogenesis of ALS. Environmental and genetic triggers that initiate these may also be triggers for step production or progression. The schematic illustrates core cellular and molecular processes observed in ALS and active during the prolonged subclinical phase Rather than acting as independent or deterministic causes of disease onset, these cascades emerge, amplify, and reinforce one another, collectively lowering cellular resilience and facilitating progression from one step to the next in the multistep process. Their activation and relative dominance vary between individuals and disease stages and are influenced by genetic susceptibility as well as a wide array of environmental modifiers, including metabolic stress, infection, inflammation, toxic exposures, and physical trauma. Once a tipping point is reached these same cascades contribute to self-propagating motor neuron degeneration and overt disease progression.

While early steps (genetics and perinatal exposure) provide disease foundation, the intermediate steps (3–6) accumulate in the background of a biological “interactome” characterized by a non-linear cluster of stressors feeding into one another [50,51]. These are not considered discrete molecular events but higher-order biological transitions, which eventually cross the threshold of cellular and network resilience causing compensatory failure. There is no single, universal “trigger” but a large array of potential traumatic and non-traumatic environmental exposures, any of which alone or in combination act as a catalyst to move one step to the next. This occurs over a long subclinical phase, during which interchangeable convergent pathological cascades interact. They include protein misfolding and aggregation, failing proteostasis, mitochondrial dysfunction, oxidative stress, neuroinflammation (microglial and astrocytic activation), and blood–brain barrier disruption (See Figure 2). They occur in variable non-determined order and interact non-linearly reinforcing one another and are modulated by environmental exposures [52,53]. It should not be inferred that the well-recognized pathogenic cascades related to ALS equate to triggers for step initiation or progression. However, it is likely that any event that can initiate or further a pathogenic factor may also be a step trigger (See Figure 2).

ALS frequently manifests focally, despite widespread genetic and environmental influences [54], and focal cortical thickness within the primary motor cortex corresponds to clinical impairments [55] This implies that one or more steps involve the emergence of regional permissiveness within motor systems and its networks driven by differences in metabolic demand, connectivity, and developmental variations [56]. It has been hypothesized that a potential mechanism underlying disease spread in ALS is through the diffusion of toxic factors in the neuron’s microenvironment [57].

### 1.5. Aging and Step Development

Later steps in the multistep process might be considered to be age and senescence related [49]. Different neuron types show distinct susceptibility to age-dependent degeneration and distinct neurons differ in their biological age [58]. Humans appear uniquely vulnerable to neurodegenerative diseases including ALS. The involved cerebral structures and molecular pathways are recent in evolutionary terms rendering them more vulnerable to insults or stressors accumulating with ageing [59,60]. Restriction of ALS and other neurodegenerations to humans may reflect a beneficial genetic architecture that evolved to expand cognition, a complex motor repertoire and sophisticated motor control in modern humans [61], but which is incompatible with modern day longevity. For example, the gene causing Huntington’s shapes neurodevelopment driving a superior brain in early life is vulnerable to accelerated aging later in life [62]. Overt clinical ALS occurs when cumulative cellular stress exceeds compensatory capacity (the “tipping point”). This might be precipitated by acute systemic stressors such as trauma, severe inflammation, infection, or toxic exposure. Trauma occurring near this threshold may abruptly reduce motor reserve, thereby unmasking preclinical disease, explaining patient narratives of ALS “starting” after surgery, falls, or injuries, despite a long preceding subclinical phase. Once the threshold is crossed, the neurodegenerative process becomes self-propagating and motor neuron loss is unrelenting [63].

Aging and ALS share biomolecular cascades [64,65,66,67]. There is a substantial loss of neuronal proteome maintenance with ageing considered be causal for age-related synapse loss and functional decline [68]. Recently four major topological turning points across the lifespan occurring around 9, 32, 66, and 83 years old have been identified [69]. Each has a distinct topological development with specific changes [69]. Although unproven, this observation might underlie new steps occurring during these select aging stages. Exceptionally long-lived individuals might have a favorable DNA methylation profile, enabling a slower rate of aging. Such individuals could still harbor subclinical ALS, but because of better compensatory biology never attain sufficient steps to cross the threshold for clinical manifestation. For example, TDP-43 aggregates, a hallmark of ALS seen in >97% of cases are present in about 25% of elderly asymptomatic individuals, a prevalence exceeding 35% in those 85 years and older [70,71].

### 1.6. Implications of the Multistep Model and Therapeutic Opportunities

The multistep model reframes ALS and probably other neurodegenerations as system-level failures unfolding over decades and integrates genetic risk, environmental exposure, cellular stress, and aging biology. During the pre-clinical period, neurons remain functionally compensated and potentially rescuable, despite accumulating proteostatic stress, mitochondrial inefficiency, axonal transport defects, and inflammatory signaling. Clinical onset indicates compensatory failure, and the degenerative process becomes self-propagating. Interrupting or slowing steps at any stage may delay disease becoming overt within a natural lifespan. Three approaches are considered, but there may be others: 1. Changing the environment, 2. Protecting early-life development, and 3. Use of ant-aging and anti-senescence therapies and strategies.

### 1.7. Changing the Environment

Through one’s lifespan, an individual encounters a wide-ranging exposure to toxicants, trauma, infection, metabolic stress, and other insults. A variety of traumas have been proposed as risk factors for ALS [72,73], including head injury [74], spinal trauma, injury associated with contact sports [75,76], military service [77], and physical activity [78]. Similarly, numerous environmental factors have been considered as risk-associated for ALS [79,80,81,82,83,84,85]. They may synergize throughout an individual’s lifetime, building an individual’s unique exposome [15]. The contribution of specific environmental toxicants has been difficult to assess, and in ALS and other neurodegenerations, null findings and recall bias have tempered the results in large-scale studies, failing to confidently define environmental risk factors [52].

Population studies have proved it difficult to assign cause–effectiveness to any specific exposure, but the multistep model accommodates combinations of any number of insults, occurring at any time, in any dose, as context-dependent risk modifiers that induce new steps or promote progression between steps. Rather than asking whether a given exposure “causes” ALS, a more appropriate question might be whether an “environment” composed of many varied factors increases the probability of acquiring one or more steps. This is exemplified by road dust [86], air and water pollution [81,87,88], micro- and nano- plastics [89], and traumatic events including surgery [90], all typically associated with urbanization and industrialization. As efforts continue to improve environmental quality, it will be important to study changes in the incidence of neurodegeneration, including ALS.

### 1.8. Protecting Early Life

**Figure 3:**
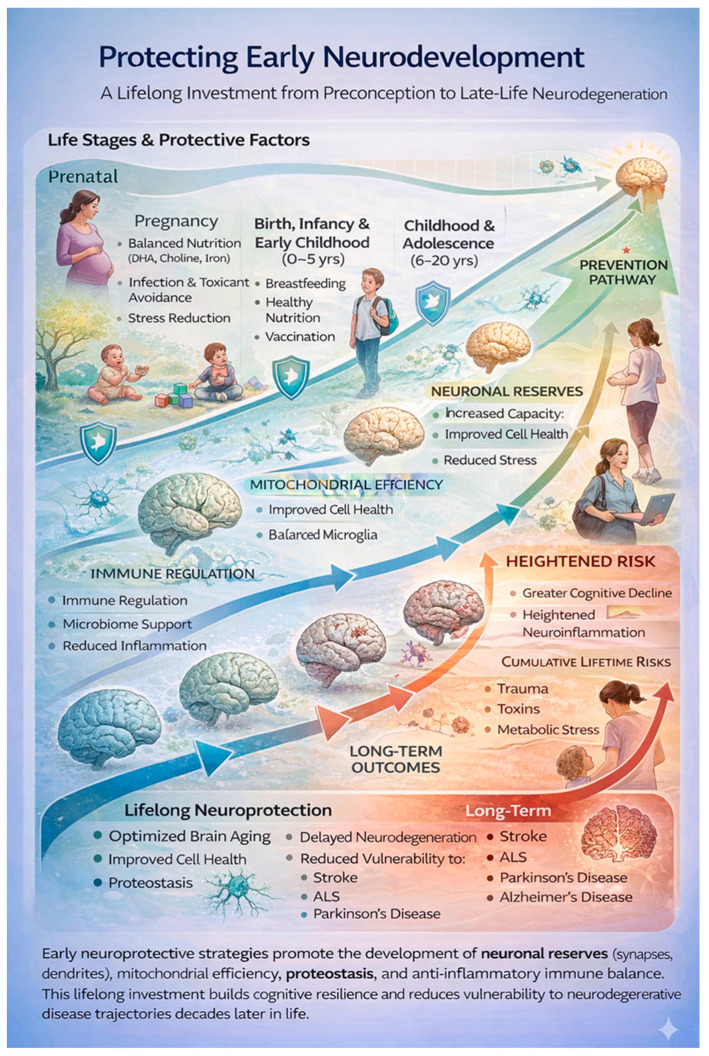
Early neurodevelopmental protection shapes late-life neurological resilience.

Pamphlett and Kullmann [46] evaluated early-life events and conditions as poly-environmental risk factors in the context of the multistep model of ALS, finding a small effect on those born and living longer in an area, having younger parents, and a lower educational attainment with fewer years of education. Exposure to adverse environmental factors during early life is step-inductive, negatively impacts neurodevelopment, and increases the risk of neurodegeneration, including ALS in later life. Steps acquired through early-life exposures may matter more than later life events, because they are associated with irreversible biological transitions. Even mild and subclinical insults during the gestational and perinatal periods are critical to neurodevelopment [91], laying the seeds for later-life neurodegeneration [92,93]. During gestation and the perinatal periods, key maturational processes, including neuronal proliferation, migration, myelination, synaptic pruning, and the finely tuned transport system essential for synapse maturation, are vulnerable to genetic, epigenetic, immune, and metabolic aberrations. Early-life microbiome dysbiosis increases blood–brain barrier permeability with potential for chronic inflammation [33]. Protecting neurodevelopment requires minimizing early biological stress, supporting nutrition and immune balance, and preserving neural resilience across the life course. Optimizing nutrition during fetal and early postnatal life is a golden opportunity to protect neurodevelopment and brain function across the lifespan [94] (see Figure 3).

Protective influences across early life, beginning in the prenatal period and extending through childhood and adolescence, build neuronal reserves, mitochondrial efficiency, immune regulation, and proteostasis. These foundational processes enhance lifelong brain resilience and reduce vulnerability and preclude STEP accumulation in the multistep model from environmental stressors, including trauma, toxins, and metabolic stress. Impaired early protection increases susceptibility to neurodegenerative diseases including ALS.

### 1.9. Strategies Mitigating Aging and Senescence

**Figure 4:**
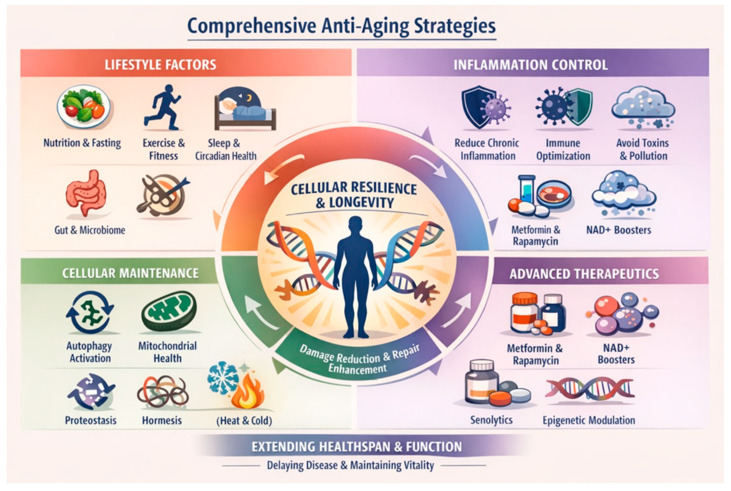
Anti-aging strategies may prevent later step accumulation so that ALS remains preclinical through a lifespan.

Later step(s) in the multistep process impact aging and senescent cell formation, and strategies mitigating these can prevent additional step formation associated with the emergence of clinical ALS [95]. Aging reflects the progressive loss of cellular resilience driven by accumulated molecular damage, impaired repair mechanisms, chronic low-grade inflammation, metabolic inflexibility, and immune dysregulation. Figure 4 summarizes the anti-aging strategies aimed to modify biological processes governed by conserved pathways, such as proteostasis, autophagy, mitochondrial health, and immune surveillance. Lifestyle interventions are the best evidence-based anti-aging strategies and adopting a lifelong healthy lifestyle characterized by moderate physical activity, combining resistance and aerobic exercise, promotes mitochondrial biogenesis, DNA repair, neurotrophic signaling, and the preservation of muscle and bone mass. Sleep is critical in regulating hormonal rhythms, immune function, and neurotoxic waste clearance through the glymphatic system. Chronic circadian disruption independently accelerates biological aging and age-related neurodegeneration [96]. Nutritional approaches with a healthy diet that maintains a normal weight and metabolic stability include adequate but not excessive protein intake, low glycemic load, and polyphenol-rich foods. Time-restricted eating may reduce oxidative stress, insulin resistance, and inflammatory signaling intake [97,98,99].

Looking forward, anti-aging medicine is shifting toward a systems-level model that integrates continuous physiological monitoring, biomarkers of inflammation and epigenetic aging [100], microbiome dynamics [101], and personalized interventions. Epigenetic clocks have demonstrated that biological aging is plastic and responsive to environmental and behavioral inputs, reinforcing the idea that aging resembles a partially reversible dysregulation rather than inevitable decay [102]. There is a growing interest in whether adult vaccines, such as the shingles vaccine, may slow biological aging beyond preventing acute infections [103]. Complimentary to anti-aging therapy is challenging cellular senescence, a stress-induced cellular state that contributes to tissue dysfunction, chronic inflammation, and a broad range of aging-associated pathologies [67]. Multiple senescence-targeted strategies have been developed, including senolytics, senomorphics, and senescence immunotherapy [104], some directed toward ALS [105]. These approaches mitigate senescence-driven pathology by eliminating senescent cells, modulating their secretory activity, or restoring cellular function.

## 2. Summary

This opinion article reframes ALS within its multistep model. It advocates for a systems-level understanding of ALS, integrating genetics, epigenetic, aging, and environmental factors. These interact over decades reducing neuronal–glial resilience culminating in symptomatic ALS. Preventive strategies, particularly targeting early-life and aging-related biology, have the potential to mitigate or reduce further step accumulation, reducing the chances of ALS becoming overtly symptomatic. Inconsistencies in epidemiological studies have arisen because they have been viewed as “causative”, rather than as environmental probabilistic modifiers of mechanistic cascades implicated in ALS pathogenesis within the multistep process. Because initial steps likely reflect early-life exposures impacting neurodevelopment, they set the stage for lifelong vulnerability permissive for later steps and resulting neurodegeneration. It is proposed that protective strategies during prenatal and early childhood periods can build neuronal reserves, reducing susceptibility to ALS and other neurodegenerations evolving throughout life. Anti-aging strategies and anti-senescent therapeutics have the potential to interrupt or delay steps induced by aging, a lifelong process, and senescent cell accumulation preventing disease emergence.

## Data Availability

There is no additional data in relation to this manuscript.
